# Attapulgite Nanorod-Incorporated Polyimide Membrane for Enhanced Gas Separation Performance

**DOI:** 10.3390/polym14245391

**Published:** 2022-12-09

**Authors:** Shiyang Zhang, Xiaochuang Lu, Mingwei Cai, Zhi Wang, Zhenjing Han, Zhiyin Chen, Rongtao Liu, Kaixin Li, Yonggang Min

**Affiliations:** 1 School of Materials and Energy, Guangdong University of Technology, Guangzhou 510006, China; 2 Institute of Environmental Research at Greater Bay, Key Laboratory for Water Quality and Conservation of the Pearl River Delta, Ministry of Education, Guangzhou University, Guangzhou 510006, China; 3 Huimai Materials Technology (Guangdong) Co., Ltd., Foshan 528200, China

**Keywords:** polyimide, attapulgite, gas separation, permeability, selectivity

## Abstract

Polyimide (PI) membrane is an ideal gas separation material due to its advantages of high designability, good mechanical properties and easy processing; however, it has equilibrium limitations in gas selectivity and permeability. Introducing nanoparticles into polymers is an effective method to improve the gas separation performance. In this work, nano-attapulgite (ATP) functionalized with KH-550 silane coupling agent was used to prepare polyimide/ATP composite membranes by in-situ polymerization. A series of characterization and performance tests were carried out on the membranes. The obtained results suggested a significant increase in gas permeability upon increasing the ATP content. When the content of ATP was 50%, the gas permeability of H_2_, He, N_2_, O_2_, CH_4_, and CO_2_ reached 11.82, 12.44, 0.13, 0.84, 0.10, and 4.64 barrer, which were 126.87%, 119.40%, 160.00%, 140.00%, 150.00% and 152.17% higher than that of pure polyimide, respectively. No significant change in gas selectivity was observed. The gas permeabilities of membranes at different pressures were also investigated. The inefficient polymer chain stacking and the additional void volume at the interface between the polymer and TiO_2_ clusters leaded to the increase of the free volume, thus improving the permeability of the polyimide membrane. As a promising separation material, the PI/ATP composite membrane can be widely used in gas separation industry.

## 1. Introduction

In the past decade, significant research progress has been achieved on polymer membrane materials for gas separation [1]. One of the largest hurdles is the permeability/selectivity trade-off, that is, permeable polymers tend to have less selectivity, and vice-versa. This trade-off is dependent on the gas diffusion mechanism of most polymeric materials [2].

Polyimide (PI) is an ideal membrane material due to its strong designability, excellent mechanical properties, heat resistance, and solvent corrosion resistance [3,4,5]. Almost all commercial polyimide membranes are made of polymers with good scalability and low cost, which are beneficial for commercial-scale gas separation. However, a key problem of polyimide membranes as a new generation of gas separation membranes is that their separation performance is limited by the trade-off effect and the Robeson upper limit is difficult to break [6,7]. Owing to the specific pore size and geometry of inorganic nanomaterials, they can be used as fillers for polyimide [8]. In addition, the structure of the polyimide membrane can be improved to overcome the upper separation limit. Silica [9,10], Titanium dioxide [11,12], molecular sieves [13,14,15], and carbon nanomaterials [16,17] have been doped into polyimide membranes and significantly improved the separation effect.

As a nanomaterial, ATP has a large specific surface area (about 200 m^2^/g), unique one-dimensional structure, low cost and strong adsorption capacity [18,19], cannot be found in other natural minerals. The reactive hydroxyl groups on the surface become compatible with the polymer matrix after proper surface modification [20]. In addition, ATP shows good gas adsorption characteristics due to large specific surface area and the crystal channels [21]. Xiang et al. [22] successfully fabricated Pebax/ATP composite membranes for CO_2_/N_2_ separation by incorporated ATP into the Pebax1657 polymer. Both the CO_2_ permeability and CO_2_/N_2_ selectivity increased at low ATP loadings (<6.3 wt%). CO_2_ permeability and CO_2_/N_2_ selectivity of the addition of 1.7 wt% ATP/Pebax composite membrane were increased respectively to 77 barrer and 52, increased by 37.5% and 30.0% compared to the pristine Pebax membrane. In addition, the ATP/Pebax composite membranes exhibited well thermal stability. Then, Xiang et al. [21] prepared a thin layer of ATP/Pebax composite membrane on a porous polyacrylonitrile (PAN) by spin coating method. The CO_2_ permeability of composite membrane was significantly increased to 108 GPU increased by 53 times, and the desired selectivity for CO_2_/N_2_ and CO_2_/CH_4_ was also improved by 35% and 16%, respectively. Mixed gas permeation measurements showed that gas permeability and selectivity decreased with the increase of feed pressure, while gas permeability increased with the increase of permeation temperature. Ahmad et al. [23] used NMP as solvent to prepared Pebax-1657/ATP composite membranes. The selectivity of CO_2_/N_2_ increased first, and then decreased with the improvement of ATP (2–10%). When the ATP content increaseed from 3% to 10%, the CO_2_/N_2_ selectivity were decreased from 69.31 to 45 due to aggregation of ATP nanorod. Wang et al. [24] prepared Carbon molecular sieving membranes from 1, 4-bis (4-amino -2-trifluoromethyl- phenoxyl benzene-1, 2, 3, 4- cyclobutane tetracarboxylic dianhydride) type polyimide incorporated with ATP. The thermal stability of carbon molecular sieving membranes was improved and the permeability was obviously increased by 3 times, however, the selectivity was slightly decreased. Therefore, the gas separation performance of polymer membrane can be effectively improved by ATP.ATP can enhance gas fluxes without reducing gas selectivity and improve the stability of polyimide membranes.

Herein, PI/ATP composite membranes were prepared using PAA as a polymer and ATP nanoparticles as a filler by in-situ polymerization that can be applied in the gas separation. ATP was intercalated into the PI polymer to increase free volume of membrane, so as to improve the gas permeability with no significant change in gas selectivity.

## 2. Materials and Methods

### 2.1. Materials

ATP was obtained from Mingguang County, Anhui Province, and γ-aminopropyltriethoxysilane (99%) and N, N-dimethylacetamide (DMAc) were purchased from Aladdin and McLean, respectively. Pyromellitic dianhydride (PMDA) and 4,4-diaminodiphenyl ether (ODA) were acquired from Changzhou Sunshine Pharmaceutical. Anhydrous ethanol and hydrochloric acid were obtained from Guangzhou Baijun Technology and Guangzhou Guangshi Reagent Technology, respectively. All the above reagents were of analytical grade.

### 2.2. Preparation of Modified ATP

The modified method of ATP was referred to the reports of previous literature [25]. In brief, 10 g of ATP was ground and sieved with a 400-mesh sieve. The sieved ATP was slowly poured into 400 mL of HCl solution with an concentration of 1 mol·L^−1^, sonicated for 30 min, and stirred at 60 °C for 8 h. The above reactants were repeatedly suction-filtered and washed until neutrality. The samples were then stored under vacuum at 80 °C for further use.

In brief, 30 g of acidified ATP was added to 500 mL of 95% (*v*/*v*) ethanol/water mixture with stirring at room temperature for 60 min. Meanwhile, 6 mL of KH-550 was hydrolyzed in a mixture of 6 mL of water and 30.6 mL of ethanol for 60 min. The hydrolyzed KH-550 was then added to the ATP slurry, and the above mixture was stirred at 70 °C for 8 h. The obtained reaction product was washed twice by suction filtration with ethanol and then repeatedly washed with deionized water until neutrality. The filter cake was dried under vacuum at 80 °C for 48 h and then ground for further use.

### 2.3. Preparation of ATP/Polyimide Composite Membrane

**Scheme 1:**
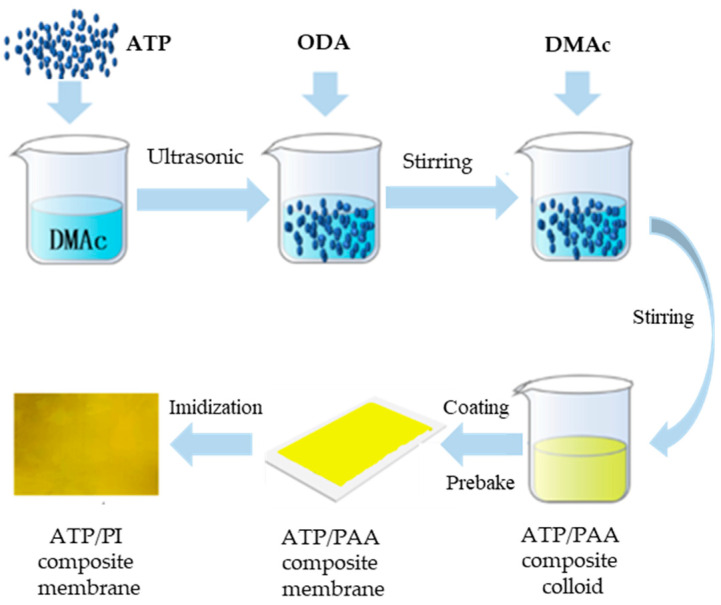
The preparation flow chart of PI/ATP composite membranes.

ATP (0, 0.60 g, 1.2 g, 1.8 g, 2.4 g, 3.0 g or 3.6 g) was added to 24.0 g of DMAc and dispersed by sonication for 1 h. Afterward, 2.8422 g of ODA was added to the ATP dispersion and stirred until the ODA monomer was completely dissolved. The above mixed solution was slowly added with 3.1578 g of PMDA and stirred at room temperature for 12 h (3500 r/min). The obtained polyamic acid (PAA) composite colloid was coated on a glass plate and then heated at 90 °C, 260 °C, and 360 °C for 30, 10, and 10 min, respectively. After the membrane was naturally cooled to room temperature, the PI/ATP composite membrane was obtained. The obtained composite membranes were denoted as PI, PI-10%ATP, PI-20%ATP, PI-30%ATP, PI-40%ATP, PI-50%ATP and PI-60%ATP, respectively. The experimental preparation of PI/ATP composite membrane is shown in Scheme 1.

### 2.4. Characterization

The functional groups of ATP, PI and PI/ATP composite membranes were characterized by a Ni-coletiS50 Fourier transformed spectrometer (FTIR, Thermo Fisher Scientific, Dreieich, Germany) in the wavenumber range of 600–4000 cm^−1^. The structures of the samples were obtained using X-ray diffraction (XRD, Max 2200, Rigaku Co., Ltd., Tokyo, Japan) with CuKα radiation (λ = 1.5406 Å) at a generator voltage of 40 kV. The morphologies of the samples were observed by a scanning electron microscope (SEM, SU8100, Hitachi Co., Ltd., Tokyo, Japan) with which the energy dispersive X-ray spectroscopy (EDS) mapping results of samples were also obtained. Thermogravimetric analysis (TGA) was carried out on a TAQ50 thermal analyzer (Mettler Toledo company, Switzerland) at heating rate of 10 °C·min^−1^ under an argon atmosphere. Coefficient of thermal expansion (CTE) of the samples were measured by the thermomechanical analyzer (TMA 450, TA Instruments, Newcastle, PA, USA).

### 2.5. Experimental Method

A custom-made gas permeation device was utilized to test the permeation properties of the membrane, and the details of the gas permeability testing device were the same as reported in the literature [26]. The membrane was sealed into the membrane cell, and the downstream of the membrane was evacuated for 12 h to eliminate all gas in the membrane, and then the upstream and downstream of the system were evacuated. The tested gas was fed into upstream and permeated through the membrane to downstream. The downstream gas pressure was measured by a vacuum flowmeter. In detail, the permeability (P) and the ideal gas selectivity (aA/B) could be calculated according to the following equations [26]:

(1) $$P=D\times S=10^{10}\times \frac{V_{d}\times l}{p_{up}\times T\times R\times A}\times \frac{dp}{dt}$$

where *P* is the permeability (barrer), *V_d_* is the calibrated permeate volume (cm^3^), *l* is the membrane thickness (cm), *p_up_* represents the upstream pressure (cmHg), *A* and *T* denote the effective membrane area (cm^2^) and the operating temperature (308 K), *R* is the gas constant (0.278 cm^3^ cm Hg cm^−3^ (STP) K^−1^) and *dp/dt* is the steady-state downstream pressure increase (cm Hg s^−1^).

(2) $$a_{A/B}=\frac{P_{A}}{P_{B}}$$

where *P_A_* and *P_B_* represent permeability of gases A and B, respectively.

## 3. Results and Discussion

### 3.1. Characterization of PI/ATP Composite Membrane

**Figure 1:**
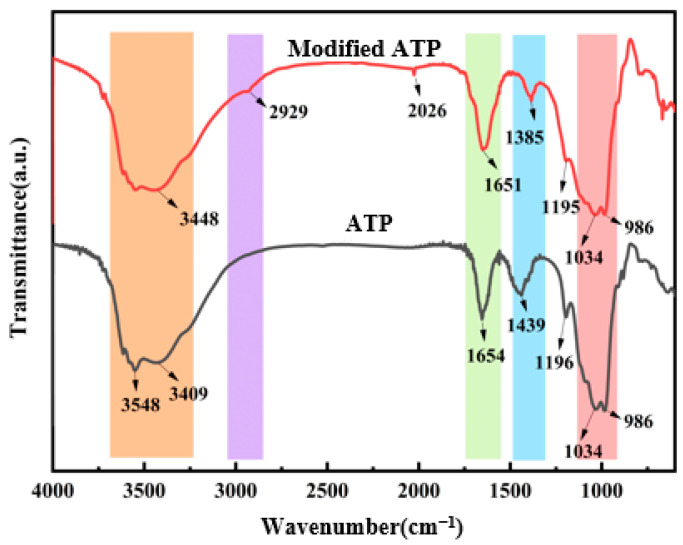
FTIR spectra of ATP before and after modification.

The functional groups of ATP before and after modification were analyzed by FTIR, as shown in Figure 1. The absorption peaks of ATP at 3409 cm^−1^, 1654 cm^−1^, 1385 cm^−1^, 1034 cm^−1^ and 986 cm^−1^ are attributed to -OH, HO, Si-OH, C-Si-O and Al (Mg)-OH groups, respectively [27]. Compared with ATP, the absorption peak of ATP-KH-550 at 3548 cm^−1^ belonging to the silyl hydroxyl group is significantly weakened, and an obvious absorption peak is appeared at 2929 cm^−1^. The bending and stretching vibration peaks of Si-O-Si are at 1115-970 cm^−1^, where the absorption peak of ATP is weak, while the ATP modified by KH-550 shows a larger and sharper absorption peak. indicating that there is a stronger covalent binding between KH-550 and ATP. KH-550 is hydrolyzed to generate silanols [28], which form hydrogen bonds with the silanols on the surface of ATP, and then the strong Si-O-Si covalent bonds is condensed at high temperature. The silanols between the molecules associate with each other to form a membrane with a network structure, which covers the surface of ATP. Compared with the unmodified ATP, the total thermal mass loss of the modified ATP increased by 7.27% (Figure S1), which further proved that KH-550 was successfully grafted on the surface of ATP.

**Figure 2:**
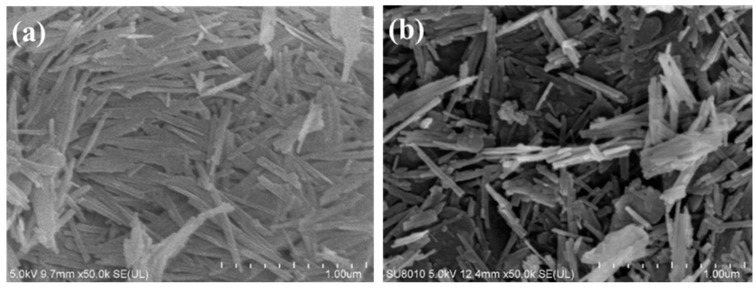
SEM images of ATP before modification (**a**) and after modification (**b**).

The SEM images before and after ATP modification are shown in Figure 2a,b, respectively. It can be seen from the figure that the ATP modified by KH-550 has better dispersibility compared with ATP. The rod crystal beams are uniformly dispersed and relatively small, and the modified ATP is more suitable as the filler of PI matrix.

**Figure 3:**
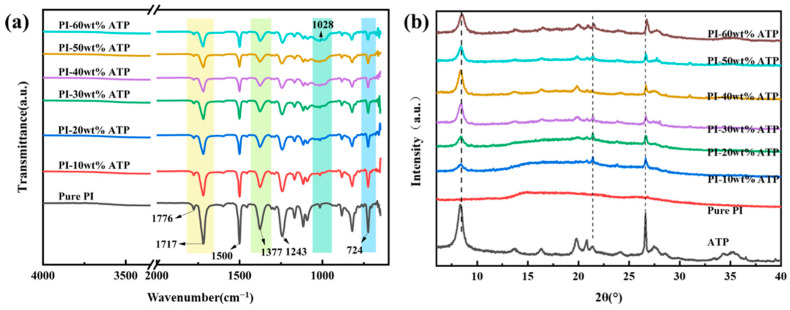
(**a**) ATR-FTIR curves and (**b**) XRD curves of pure PI and ATP / PI composite membranes.

Figure 3a shows the FTIR spectra of PI and PI/ATP composite membranes. The characteristic peaks of PI at 1776, 1717, 1500, 1377, 724 cm^−1^ are attributed to C=O symmetric and asymmetric stretching of the imide group [29], C–N stretching of the C–N–H group [30], C–N [31] and C=O bending [32]. Compared with the absorption peaks of ATP (Figure 1) and pure PI membrane, the absorption peak of PI/ATP composite membrane shows the characteristic peaks of ATP and PI membrane structure, and the change of absorption band is not obvious. Furthermore, the intensity of the absorption peak at 1028 cm^−1^ increased as the ATP loading increased from 10 wt% to 60 wt%, indicating the presence of a higher amount of ATP in the PI matrix. Figure 3b exhibits the XRD results of ATP, PI, and PI/ATP composite membranes. ATP presents a strong diffraction peak at 8.2°, which corresponds to the (110) [33] crystal plane of ATP. This finding indicates that the structure of APT is preserved through acidification and modification [34]. For the composite, its diffraction peaks include a 2θ of 8.2°, and the peak intensity at 2θ = 8.2° generally increases with the APT content. This result implies that the nucleation effect of APT nanorods plays a major role in crystallinity [35].

**Figure 4:**
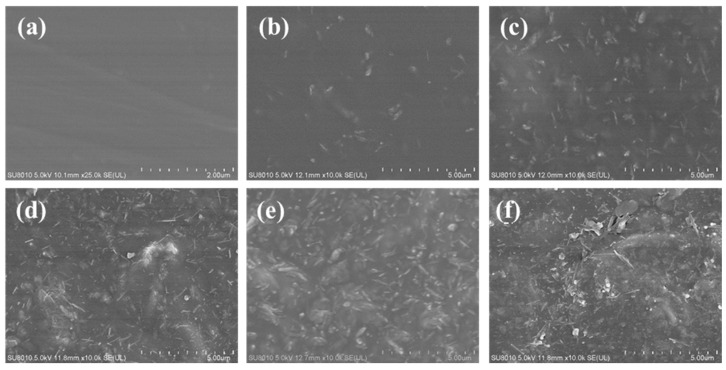
SEM images of the surface of membranes: (**a**) PI, (**b**) PI-10% ATP, (**c**) PI-30% ATP, (**d**) PI-40% ATP, (**e**) PI-50% ATP, and (**f**) PI-60% ATP.

The surface morphologies of the PI/ATP composite membranes were observed by SEM. The diameter of the ATP nanorods is only 30 nm, which allows the easy formation of a structure consisting of the inorganic phase ATP fully covered by the organic phase PI. Figure 4 presents the surface SEM images of the PI/ATP composite membranes with different ATP contents. The pure PI membrane has a uniform surface. Figure 4b–e show that despite the gradual increase in the doping amount of ATP rod-like particles, they are always uniformly distributed in the PI membrane. No large interfacial cavities are observed, and the composite phase can be well formed, thereby establishing nanochannels for gas (H_2_, He, N_2_, O_2_, CH_4_, and CO_2_) permeation. However, excess nanoparticles particles easily aggregate, which is unfavorable to the overall separation performance of the membrane [36]. At 60 wt% loading, ATP shows evident agglomeration (Figure 4f). the inorganic nanoparticles could not be well dispersed in the polymer and the compatibility with the polymer matrix was reduced [11]. Defects appear in the membrane, resulting in poor gas selectivity of the membrane.

**Figure 5:**
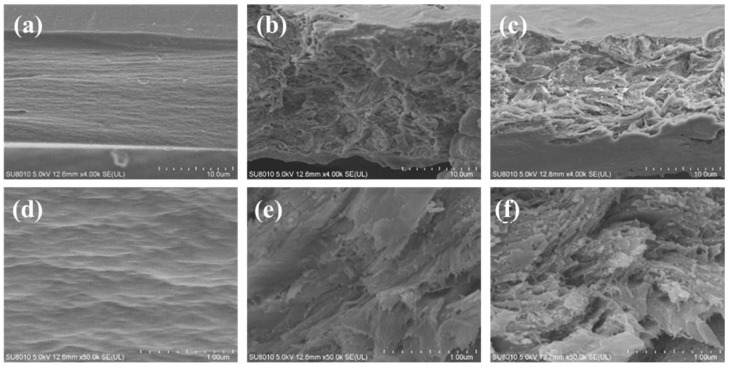
SEM images of cross section of membranes: (**a**,**d**) PI, (**b**,**e**) PI-50% ATP, (**c**,**f**) PI-60% ATP.

**Figure 6:**
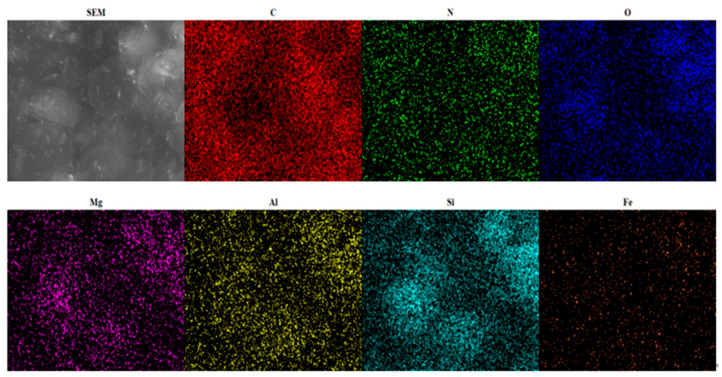
Elements of the surface of distribution of PI-50% ATP composite membrane.

The cross-sectional SEM images of pure PI, PI-50%ATP, and PI-60%ATP composite membranes were observed to further study the recombination of nanorod-like structure ATP in PI membranes as shown in Figure 5a–f. Figure 5a displays that the pure PI membrane has a dense structure and a relatively smoothly fractured surface. This is consistent with the reported cross-sectional morphology of polyimide membranes [37]. As shown in Figure 6b,c, small fragments were appeared at the PI cross section fracture, which may be due to induced cracks because of the increases of free volume fraction at higher ATP contents [38]. the nanorod-like ATP is uniformly distributed in the PI organic phase (Figure 6b). The ATP inorganic phase and the PI organic phase are well combined and have formed nanochannels, which can improve permeability without reducing gas selection. Figure 6c reveals that the ATP particles are partially aggregated in the membrane due to the excessive amount of ATP, and gas can permeate directly inside the agglomerated ATP. This result is consistent with the SEM image of the membrane surface.

Figure 6 and Figure S1 are the element distribution diagrams of the surface and cross-section of the PI-50%ATP composite membrane, respectively. C, N, O, Na, Mg, Al, and Fe elements can be found in the surface and cross-section of the PI/ATP composite membrane. The main elements of ATP (Na, Mg, Al, and Fe) [39] and PI (C, N) are included, and their distribution is relatively uniform. This finding further verifies that ATP and PI are well combined.

**Figure 7:**
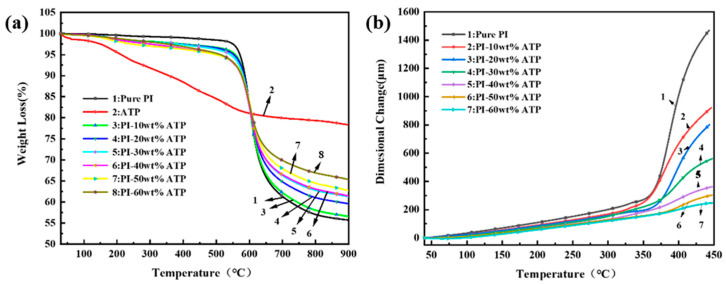
(**a**) TGA curves (**b**) TMA curves of pure PI and ATP/PI composite membranes.

The thermogravimetric properties of PI/ATP composite membranes were investigated by TGA as shown in Figure 7a. It can be clearly seen that there is a small amount of mass loss of powdered ATP during the whole pyrolysis process, and the residual weight is more than 75%, indicating that the ATP has a high thermal stability [40]. Compared with that of the pure PI membrane (571.3 °C), the thermal stability of the PI/ATP composite membrane decreases slightly. When the ATP content is 50% and 60%, the T5 of the PI/ATP composite membrane is 499.6 °C and 491.6 °C, respectively. This result can be attributed to the presence of adsorbed water, zeolite water, crystal water, and structural water in ATP. These types of water volatilize when the temperature increases. However, the T_30_ of the composite membranes increases with the ATP content. When the ATP content is 50%, the T_30_ can reach 666.1 °C, which is higher than that of the pure PI membrane (629.7 °C). Overall, the PI/ATP composite membranes can be used to separate gases at high-temperature stages.

Figure 7b shows the TMA curves of pure PI and PI/ATP composite membranes, and Table S1 displays their CTE values. With the increase in the ATP content, the CTE of the membranes decreases significantly. When the ATP content is 60%, the CTE value of the PI/ATP composite membrane is 25.4 ppm/k, which is 41.1% lower than that of the pure PI membrane (43.1 ppm/k). The strong interaction between ATP with extremely low CTE value and the PI matrix is provided by the coupling agent KH-550, which enables the ATP particles to bind to the polymer chains. During heating, the expansion of the polymer chains can be stopped by the ATP rods. The CTE of the composite membrane is small, thus reducing the change of gas separation performance at high temperature.

### 3.2. Pure Gas Separation Performance

**Figure 8:**
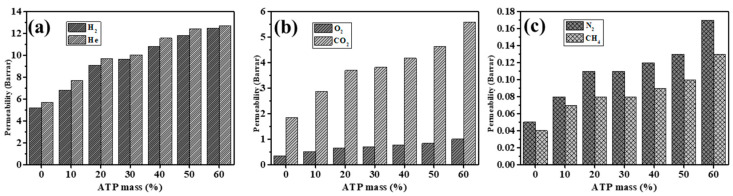
Gas permeability performance (**a**) H_2_, He, (**b**) N_2_, CH_4_, and (**c**) O_2_, CO_2_ of PI/ATP composite membranes with different ATP content.

The gas separation performance of PI/ATP composite membranes was evaluated according to their permeability and selectivity. Figure 8 shows the effect of ATP content on the gas permeability of PI composite membranes. Figure 8a–c show that when the content of ATP is 60%, the gas permeation of H_2_, He, N_2_, O_2_, CH_4_, and CO_2_ is 12.51, 12.70, 0.17, 1.00, 0.13, and 5.59 barrer, respectively, which are increased by 140%, 124.0%, 240%, 185.7%, 225% and 203.8%, respectively, compared with those in pure PI (5.21, 5.67, 0.05, 0.35, 0.04, and 1.84 barrer, respectively). The enhanced gas permeability of the PI/ATP composite membrane is mainly attributed to the change of free volume and void upon addition of ATP nanorod. The interaction between polymer chain stacking is disrupted by ATP in the polymer matrix by expanding the free volume in the membrane, which changes the structural law of the nano particle polymer interface. In addition, Interface pores and voids may be formed due to the weak compatibility of polymer and inorganic nanoparticles at the interface and agglomeration of inorganic nanoparticles [41,42]. Therefore, ATP enhances the gas diffusivity of PI, thereby improving the separation performance of the PI/ATP composite membrane.

Table 1 presents the effect of ATP content on the gas selectivity of the PI composite membrane. When the ATP amount is 10–50%, The selectivity of gas generally decreases and the segregation increase with the increase in the ATP content. When the doping amount is 60%, the selectivity of CO_2_/CH_4_, O_2_/N_2_, and H_2_/CH_4_ decreases from 46.40, 6.46, and 118.20, respectively, to 43.00, 5.88, and 96.23, respectively, compared with those under 50% dosage. When the dosage is 60 wt%, the ATP nanorods are poorly dispersed in the polymer phase and exhibit agglomeration. Owing to the pinholes generated by the agglomeration of ATP particles, nonselective defects are formed in the resulting composite membranes [43]. This phenomenon is especially serious when the loading of ATP in the composite membrane continues to increase. As a result, the gas separation performance of the membrane decreases. Therefore, when the content of ATP was 50 wt%, the gas permeability and selectivity of the PI/ATP composite membrane exhibited an optimal balance, which was superior to many reported common polymer membranes, as summarized in Table 2.

**Figure 9:**
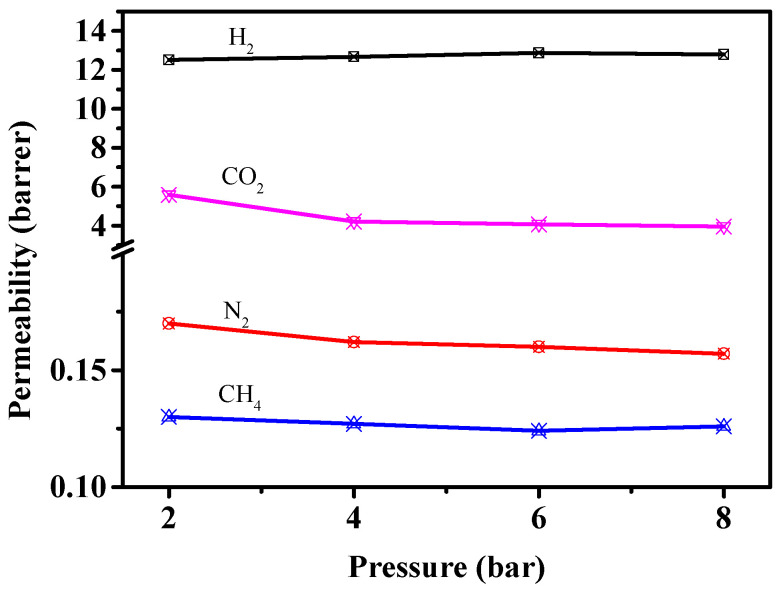
H_2_, N_2_, CH_4_ and CO_2_ pure gas permeability of PI-50 wt% ATP composite membranes at different gas pressures.

The gas separation experiment of PI-50 wt% ATP at 2–8 bar was also further carried out. The gas penetration behavior of pure PI membrane and PI/ATP composite membrane are shown in Figure 9. It can be seen from Figure 9 that the permeability of the PI-50 wt% ATP composite membrane for H_2_, N_2_ and CH_4_ remained nearly unchanged while the permeation flux of CO_2_ is significantly reduced with the increase of pressure, which is consistent with the relevant reports [49]. When the gas pressure increases to 8 bar, the permeability of H_2_, N_2_, CH_4_ and CO_2_ are 12.79, 0.157, 0.126 and 3.96 barrer respectively, and the permeability of CO_2_ decreases by 29.16% with pressures ranging from 2 bar to 8 bar. This result is consistent with the gas transport dual-mode transport model. The Langmuir model is related to the free volume formed in the glassy state. When the pressure increases to a higher level, the Langmuir site will be saturated, resulting in a decrease in permeability [50,51]. When the pressure is increased to 8 bar, no obvious change in the selectivity of H_2_/N_2_ and H_2_/CH_4_ were also observed (Table S2). Therefore, PI/ATP composite membranes can be used to separate gas at higher pressure.

## 4. Conclusions

In this work, PI/ATP composite membranes with different amounts of organically modified ATP were prepared by in-situ polymerization. During the preparation of polyimide composite membrane, the content of ATP played a major role on the membrane structure and gas separation performance. Due to the poor compatibility between ATP surface and PI, the structure of the membrane is strongly affected by ATP nanorods, leading to the formation of nano channels in the polyimide matrix. These channels are sensitive to certain dynamic diameters, providing a channel for gas penetration. When the ATP content was 50%, good balance between permeability and selectivity was achieved. The gas permeability of the PI/ATP composite membrane increased remarkably, and gas selectivity did not decrease significantly. PI/ATP composite membrane can also be applied at higher temperature and pressure. Inexpensive ATP can be effectively compounded with polyimide, thus providing a new method to prepare PI-based composite membranes for gas separation. PI/ATP composite membrane was a promising new type of gas separation membrane.

## Figures and Tables

**Table 1:** Selectivity of pure polyimide and nano ATP polyimide composite membranes.

Membrane	CO_2_/CH_4_		O_2_/N_2_		H_2_ /CH_4_	
	Selectivity	Segregation Index	Selectivity	Segregation Index	Selectivity	Segregation Index
Pure PI	46.00	1.84	7.00	0.35	130.25	5.21
PI-10 wt% ATP	47.83	2.87	6.50	0.52	113.83	6.83
PI-20 wt% ATP	46.25	3.7	6.00	0.66	113.63	9.09
PI-30 wt% ATP	47.75	3.82	6.46	0.71	120.63	9.65
PI-40 wt% ATP	46.44	4.18	6.50	0.78	120.22	10.82
PI-50 wt% ATP	46.40	4.64	6.46	0.84	118.20	11.82
PI-60 wt% ATP	43.00	5.59	5.88	1	96.23	12.51

**Table 2:** Gas separation property comparison between some membranes reported in literatures and this study.

Membrane	Permeability (Barrer)			Selectivity		Ref
	H_2_	CO_2_	H_2_/CH_4_	CO_2_/CH_4_	O_2_/N_2_	
PI-0.2 wt% TAP		0.194		87		[14]
PSF-0.2 wt% SiO_2_		19.7		18	4.5	[36]
PVA-40 wt% TiO_2_					1.84	[44]
PC-10 wt% MWNTs		12.79		30.45		[45]
Pebax-19 wt% SiO_2_					3.2	[46]
Matrimid^®^-10 wt% Cu	16.91		69.15			[47]
Pebax-2 wt% MWNT					2.1	[48]
PI-50 wt% ATP	11.82	4.64	118.20	46.40	6.46	this work
PI-60 wt% ATP	12.51	5.59	96.23	43.00	5.88	this work

## Supplementary Materials

The following supporting information can be downloaded at: https://www.mdpi.com/article/10.3390/polym14245391/s1, Figure S1: TGA curves of ATP before and after modification; Table S1: Main parameters of thermal properties of pure PI and PI/ATP composite membranes; Table S2: Selectivity of PI/ATP composite membranes at different gas pressures.
