# Supplementary material for: Attapulgite Nanorod-Incorporated Polyimide Membrane for Enhanced Gas Separation Performance
Source: Polymers (Basel). 2022 Dec 9;14(24):5391. doi: 10.3390/polym14245391 (PMC9785766; doi:10.3390/polym14245391)
Supplement: Supplementary file 1 [file polymers-14-05391-s001.zip › polymers-2034324-supplementary.pdf]

polymers

Supporting information

Attapulgite Nanorod-Incorporated Polyimide Membrane for Enhanced Gas Separation Performance

Shiyang Zhang [1], Xiaochuang Lu [1], Mingwei Cai [1], Zhi Wang [1], Zhenjing Han [1], Zhiyin Chen [1], Rongtao Liu [2,*], Kaixin Li [1] and Yonggang Min [1,3,*]

[1] School of Materials and Energy, Guangdong University of Technology, Guangzhou 510006, China; syz131139@163.com (S.Z.); lxc5200816@163.com (X.L.); 2112102037@mail2.gdut.edu.cn (M.C.); zhiw8_15@outlook.com (Z.W.); m18320314261@163.com (Z.H.); czy030215@icloud.com (Z.C.) li.kx@gdut.edu.cn (K.L.)
[2] Institute of Environmental Research at Greater Bay, Key Laboratory for Water Quality and Conservation of the Pearl River Delta, Ministry of Education, Guangzhou University, Guangzhou 510006, China
[3] Huimai Materials Technology (Guangdong) Co., Ltd., Foshan 528200, China
* Correspondence: liu44101@163.com (R.L.); ygmin@gdut.edu.cn (Y.M.)

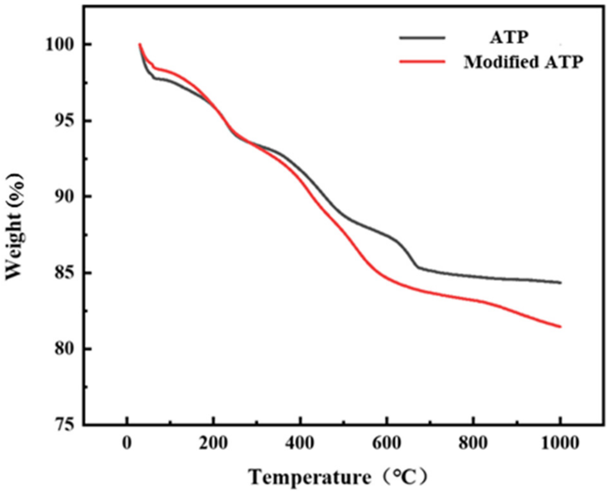

Figure S1. TGA curves of ATP before and after modification

Table S1. Main parameters of thermal properties of pure PI and ATP/PI composite films

Samples	T_g/°C	$T_{5\%}$/ °C	$T_{10\%}$/ °C	$T_{30\%}$/ °C	R_{w850} °C	T_{max}/ °C	CTE1 /ppm	CTE2 /ppm	CTE3 /ppm
ATP	-	212.9	357.5	-	78.9	421.8	-	-	-
Pure PI	368.1	571.3	586.7	629.7	56.2	600.9	43.1	48.7	220.1
PI-10wt% PI	369.4	557.5	583.8	631.8	57.2	602.8	36.5	41.2	135.8
PI-20wt% PI	374.9	553.0	583.9	641.7	60.2	602.9	33.6	38.2	115.6
PI-30wt% PI	375.5	542.8	581.5	650.3	62.0	603.1	31.3	37.7	83.03
PI-40wt% PI	376.2	502.3	576.1	650.7	62.2	603.4	27.0	32.5	54.4

PI-50wt% PI	379.8	499.6	578.7	666.1	63.6	603.6	26.2	31.3	45.0
PI-60wt% PI	381.9	491.6	576.8	696.5	66.1	601.9	25.4	30.7	39.0

Table S2. Gas selectivity of ATP/PI composite membranes at different gas pressures

Pressure (bar)	CO_2/CH_4	CO_2/N_2	H_2 /CH_4	H_2/N_2
2	43	32.88	96.23	73.59
4	33.15	25.98	99.76	78.21
6	32.825	25.43	103.79	80.43
8	31.43	25.22	101.51	81.46
